# A Social Norms Analysis of Religious Drivers of Child Marriage

**DOI:** 10.9745/GHSP-D-23-00339

**Published:** 2024-04-29

**Authors:** Olivia Wilkinson, Kerida McDonald, Susanna Trotta, Jennifer Philippa Eggert, Florine de Wolf

**Affiliations:** a Joint Learning Initiative on Faith and Local Communities, Washington, DC, USA.; b UNICEF, Jamaica.; c Joint Learning Initiative on Faith and Local Communities, Genoa, Italy.; d Joint Learning Initiative on Faith and Local Communities, Birmingham, United Kingdom.; e Joint Learning Initiative on Faith and Local Communities, Brussels, Belgium.

## Abstract

Social norms theory provides a useful analytical structure for understanding religious influences on drivers of child marriage. This framework can be used to examine religious influences on child marriage as part of contextual analysis for social and behavior change interventions.

## Key Messages

- Social and behavior change interventions to reduce child marriage must address root causes to resolve deep-seated social and cultural issues that drive child marriage.
- Synthesizing research on faith actors in global health and development and viewing these research results through the lens of social norms theory, this article puts forward a framing for analyzing religious drivers of child marriage.
- This framework can help practitioners better understand the root causes of child marriage when designing social and behavior change interventions that aim to work with faith actors on religiously related drivers of child marriage.
- This tool is designed for use during contextual analysis and formative research phases of program design.

## INTRODUCTION

This article provides an analysis of social and behavioral drivers of child marriage to advance the protection and empowerment of girls from child marriage. Child marriage directly negatively affects girls and boys and, more broadly, negatively impacts their families and communities. Our analysis emerges from work undertaken for UNICEF’s Global Initiative on Faith and Positive Change for Children, Families, and Communities (FPCC). Child marriage affects many dimensions of a girl’s well-being, from their health to their education. Every year, 12 million girls^1^ across countries, cultures, religions, and ethnicities experience child marriage, which, according to UNICEF’s definition, is understood as “any formal marriage or informal union between a child under the age of 18 and an adult or another child.”^2^ Delaying marriage improves girls’ opportunities to attend school, select a safe and healthy livelihood and “develop more fully as an individual in her own right.”^3^ Child marriage is a persistent problem despite the fact that many countries with high rates of child marriage have civil laws that explicitly prohibit child marriage and set a minimum age for marriage.^4^

Faith actors are both a force in perpetuating child marriage and a key potential ally for discouraging the practice. Different reviews have found religious influence to be both “one of the strongest of all major causes of child marriage”^5^ while also “caution[ing] against broad generalizations about child marriage and faith affiliation… [because of] substantial heterogeneity within the adherents of any particular faith as to how the practice of child marriage is considered.”^5^ Within the broader discourse of women and girls’ human rights, “seeking contact with [religious] leaders…might be the most important strategic instrument.”^6^ While interventions, such as cash transfers,^7^ can significantly reduce child marriage, such interventions do not directly address the underlying social norms that promote this practice. Therefore, it is strategically important to design interventions that affect those norms and potentially promote larger impacts through faith-led, community-based initiatives.

Research on religions as they relate to global health and development goals is a growing field^8^ with some discussion of child marriage already^9^^,^^10^ but no analysis explicitly through the lens of social norms theory. This article is based on a review of 28 purposively selected program reports, policy briefs, and academic articles on child marriage and religious and traditional influences and, as such, is a review and synthesis of major work in this area. We selected sources from a literature review^11^ on faith and social behavior change programming conducted by the FPCC initiative. The extensive literature review aimed to collect sources from around the world. However, searches were limited to English language sources only. More than 1,600 resources were found initially, then whittled down to 91 relevant sources, from which we selected those specifically on child marriage. We also added new sources published since the literature review from additional searches along the same parameters, bringing the sources to 28 in total.

The original contribution of this article is to purposefully use social norms theory to examine the religious drivers of child marriage as they intersect with other social norms that drive this behavior. We expect this analysis to be particularly helpful for those doing a context analysis of the intersecting factors leading to child marriage in a country context with a view to planning for implementation of a program or project that engages faith actors to reduce child marriage.

We purposefully use social norms theory to examine the religious drivers of child marriage as they intersect with other social norms that drive this behavior.

## FRAMEWORK FOR ANALYSIS

This article builds on social norms theory and research,^12^ research on child marriage and social norms,^13^ the ACT framework developed by Drexel University with UNICEF and UNFPA,^14^ UNICEF’s working definitions of social norms,^15^ and UNICEF technical guidance on tackling social norms in social and behavior change programming.^16^ We identify 4 areas around which the behavioral drivers can be grouped for greater conceptual clarity and to inform appropriate social and behavior change approaches (Box). While closely aligned, we give the caveat that the 4 areas identified do not represent UNICEF’s official definitions (see UNICEF’s working definitions document cited earlier). These 4 areas identify the descriptive and injunctive norms at play, as well as other behavioral drivers (access to information, sanctions, and benefits) that will influence religious actors’ roles in child marriage. We recognize that there is academic debate about definitions and usage of terms^14^ (for example, empirical and normative expectancies for descriptive and injunctive norms, respectively). We put forward this framing primarily to streamline some of this debate and create a practically accessible and usable diagram to help practitioners analyze religious influences in child marriage.

BOX: Framework of Social and Behavioral Drivers of Child Marriage^14^

**Access to information** refers to the drivers related to inadequate education and awareness about the consequences of child marriage on  children.

* I do because I am not fully aware of the harmful consequences.*

**Descriptive norms** are “operationalized as perceptions about what other people do.”

* I do because I think most other people do.*

* I do because that’s what people do.*

**Injunctive norms** are “operationalized as beliefs about what others approve of or think people should do.”

* I do because that’s what other people expect me to do.*

**Sanctions and benefits/outcome expectancies** are “perceived social benefits and sanctions for enacting a particular behavior.”

* I do because I fear exclusion and want social rewards and acceptance.*

* I do because there is no legal pressure against it.*

* I do for economic reasons.*

In the following section, these drivers will be narratively described in relation to their religious aspects.

## SOCIAL AND BEHAVIORAL DRIVERS OF CHILD MARRIAGE RELATED TO RELIGIONS

The Figure shows the main social and behavioral drivers of child marriage related to religions, according to the associated drivers and level(s) of influence (individual, family, faith community, wider community, and society). The rest of this section provides narrative descriptions of the points summarized in the diagram. While each of the drivers and levels are presented separately, they are closely and complexly interrelated.

**FIGURE:**
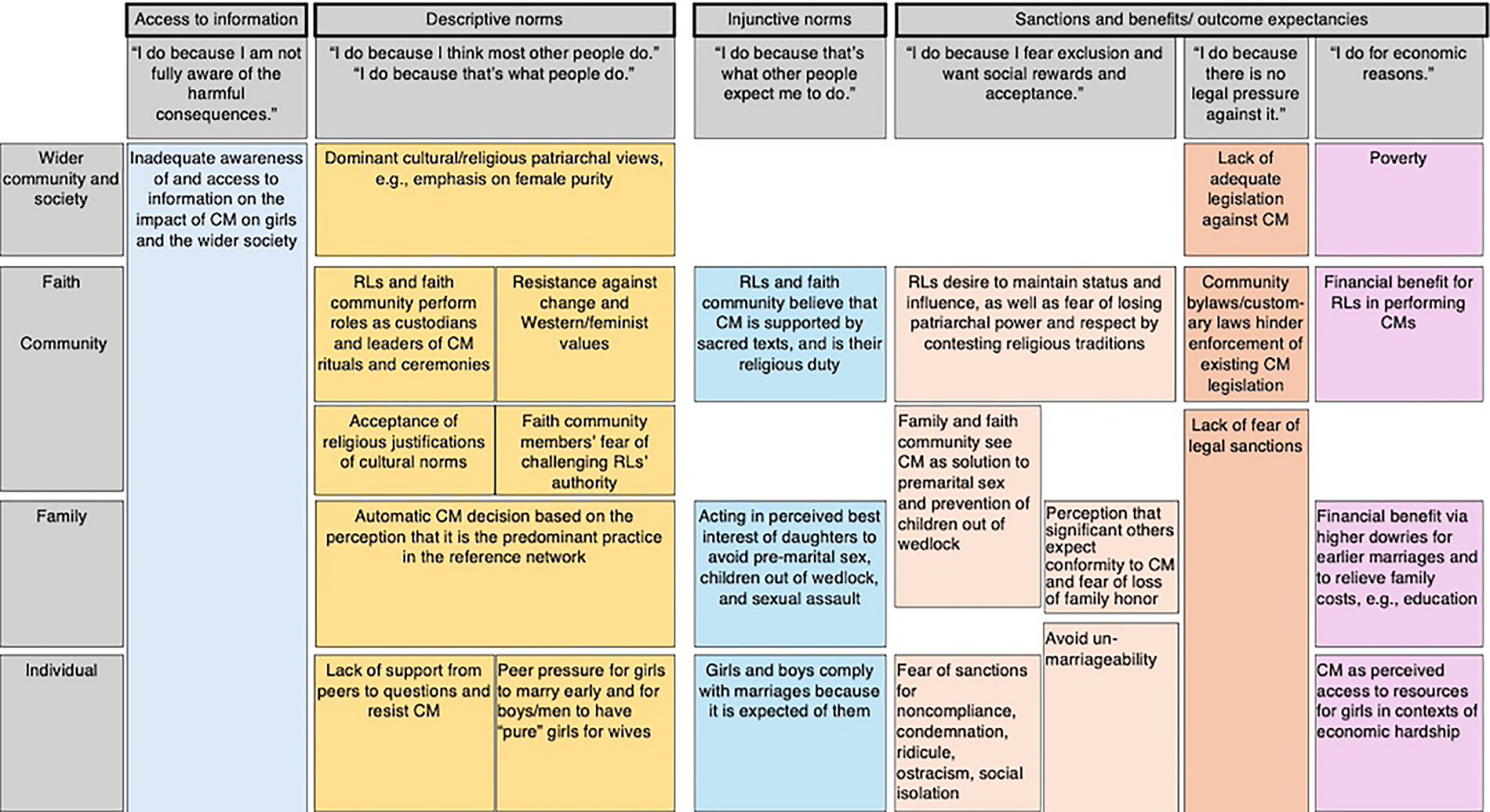
Diagram to Demonstrate the Main Social and Behavioral Drivers of Child Marriage in Relation to Religions Abbreviations: RL, religious leader, CM, child marriage.

### Inadequate Access to Information Across All Levels of Society

The lack of access to information about the laws against and impact of child marriage is a key issue at all levels. Parents, girls to become child brides, boys to become husbands, family, and community members, as well as the wider society, in some cases, have not been exposed to information or helped to consider the harmful repercussions of child marriage. Access to information is often connected to faith actors. For example, in Kenya, “community leaders (mainly chiefs, village elders, teachers, and religious leaders) are the most important source of information on [female genital mutilation] and child marriage, as reported by 24% of all respondents surveyed.”^17^ However, even if they are seen to be an important source of information, religious leaders may be unaware of the multiple repercussions for girls who enter child marriage and the related seriousness of the situation. It is also important to realize that “while faith leaders may be theologically trained, they may often lack basic knowledge about sexual and reproductive health and rights.”^18^ Moreover, sex is often avoided as a subject of conversation, so topics related to early pregnancy and/or child marriage are taboo. Religious leaders especially may see religious spaces as inappropriate to discuss matters related to sex and sexuality.^19^

### Descriptive Norms

On the family level, automatic decisions for child marriage may be based on the perception that it is a predominant practice in the reference network (“people that matter to us,” which can include a faith community network). Marrying a child can be experienced as an obvious decision to take at the family level if it is perceived as a predominant practice in the family’s close network. Under the phenomenon of pluralistic ignorance, “people may privately disdain but publicly support a norm.”^20^ For this reason, publicizing intent or commitment to change within a faith community network would be a key step in social norms programming to demystify wrong assumptions. On the individual level, a lack of support from peers to question and resist child marriage can play a decisive role. Individuals might hesitate to question or critically engage with child marriage as a perceived standard behavior in their faith community, also linked to the driver of faith community members fearing a challenge to religious authorities.

On the wider community and societal level, underlying beliefs embedded in patriarchal societies can cause community members to hold expectations for themselves and others that encourage child marriage. Disregard for female consent, the notion that only men possess the ultimate authority in making decisions for their daughters, the glorification of female purity, and condemnation of premarital sex also reflect “a complex entanglement of patriarchy and religion.”^9^ At the faith community level, religious leaders contribute to child marriage by performing the actual rituals and ceremonies as part of their role as custodians of religious and cultural traditions. For example, “patterns of patriarchal and parental authority are generally strong features of all ancient texts and when these are taken literally as unchanging ethical endorsements by God … religious leaders see their key identity and role as protecting this absolute authority of the sacred texts and tradition, and therefore can become defensive and resist any attempt that is seen by them as challenging this.”^9^ A study in 2019 on traditional practices in Malawi found that 25% of all child marriages reported are religious marriages.^21^

Efforts to end child marriage can be interpreted by some faith actors as intending to undermine a religion or a culture. Such characterizations can make communities, especially those who equate traditional practices with religious ones, more resistant to social change. The association “between child marriage and beliefs around tradition or family honor can lead to child marriage being used as a symbolic practice, and any attempts to change it may be pointed out as against religion”^9^ or as an attempt to impose “Western” and/or secular values. For example, it has been argued that “within certain contexts, faith-based legitimization of some [harmful practices] have emerged or been promoted in reaction to colonialism, or in reaction to what is experienced as Western imperialism in postcolonial contexts.”^18^

Efforts to end child marriage can be interpreted by some faith actors as intending to undermine a religion or a culture.

There is often a mingling of religions and cultures that can be difficult to untangle. In some cases, people might equate child marriage customs with religious traditions and beliefs when they have other cultural roots.^22^ Many widely accepted religious justifications for harmful practices “are actually cultural norms that have been couched in religious terminology.”^18^ Religious leaders themselves can also struggle to parse the differences between cultural and religious reasoning.^23^

### Injunctive Norms

Religious leaders and other faith actors may feel a sincere religious obligation, upheld by their interpretations of religious teachings, to support (any type of) marriage because they believe that it is an important part of the congregants’ religious lives. In many traditions, marriage is viewed as a “religious duty for all its followers and can mean that religious leaders are reluctant to put any barriers in the way of people fulfilling it, regardless of their age or the law.”^9^ Intertwined with this, in many religions and cultures, expressing sexuality outside of marriage is discouraged. Child marriage can thus be seen as a way to avoid premarital sex and children out of wedlock. For example, a challenging study in Indonesia contends that young people and their families use child marriage as “a way to manage their romantic and sexual relationships within the customary normative system.”^24^ This demonstrates family and individual agency to create solutions to meet the normative expectancies of their reference group.

### Sanctions and Benefits

#### Fear of Social Exclusion and Desire for Social Rewards and Acceptance

Religious leaders can have personal interests in maintaining their influence, income, and respect. According to many religious traditions, religious leaders are specially ordained with an exclusive power concerning the religious rite of marriage. Some may want to keep their perceived “monopoly over marriage, which includes internally consulting families on the age of the girl bride. Some religious leaders may therefore contest efforts to end child marriage … because they want to keep the meaning-making power currently held by religion and not hand it over to external secular powers.”^9^ The powers conveyed by patriarchy and religious structure on male religious leaders have a strong influence. Even more, at times, religious leaders do not want to risk losing trust in a community by opposing child marriage or other harmful practices.^25^ Consequently, they may consider “silence as the safer or easier option.”^18^ At the family and individual levels, as with injunctive norms, the drive to fit in with normative expectations is strong so that they are not excluded from their reference group because of children out of wedlock, for example. Concepts of family honor and fear of un-marriageability drive parents and children to see child marriage as a solution to avoid social isolation.

#### No Legal Pressure Against It

Legislation can be inadequate in terms of who is punishable, at what age girls and boys can legally marry, and what the sanctions are for those who are involved in the marriage. At wider community, society, and faith community levels, it has been documented that there is often a “lack of effective enforcement of existing and adequate legislation prohibiting these practices [i.e., child marriage and female genital mutilation/cutting] due to support by existing customary, traditional or religious norms which are deeply rooted.”^17^ Sometimes leaders are not “aware of the existence and consequences of a national law forbidding child marriage in their country.”^9^ Even when there are laws and people know about the law, they might not have heard of or seen cases of law enforcement. Religious and traditional norms lowering the minimum age for marriage, the lack of trust in the national justice system, the lack of hard and soft legal infrastructures, and the lack of knowledge about national legislation have been found to hinder the implementation of laws against child marriage.^17^ As a result, in communities that have been somewhat exposed to anti-child marriage campaigns or programs, “given the community attention, some parents started to hide early marriages by conducting it in secret or at night.”^26^ If legislation against child marriage is not effectively implemented, families and individuals can keep acting according to customary, including religious, norms that “entrench ideas and help give them the sense of being natural and part of the way things are or should be.”^27^

#### Economic Reasons

Poverty is an important driver of child marriage. In many contexts, child marriage is an economic decision in the face of scarcity. Families marry their children to provide for them, and religious leaders may be incentivized by financial benefits in performing child marriages. They can fear losing economic advantages connected to officiating child marriages. In some communities, they may receive fees, donations, or presents as part of their role.^28^ For example, research in Lebanon found that “regarding [child marriage] the majority felt that factors other than religion were the key causal factors. [Child marriage] was seen as existing, in most cases, due to economic considerations. Thus, while ‘religion is important, … at the end of the day you’re talking about basic needs,’”^29^ as an interviewee put it. This demonstrates how religious institutions and religious leaders can be influenced by other factors, such as religious leaders’ decisions to perform child marriages for their own or others’ financial gain in times of scarcity.

## UNDERTAKING SOCIAL NORMS ANALYSIS OF RELIGIOUS DRIVERS OF CHILD MARRIAGE

A vital first stage in conducting a social and behavioral change intervention with faith actors is to properly understand the drivers behind the behaviors, understanding that the religious aspect of these drivers may be complexly interwoven with other social, cultural, political, and economic aspects. The diagram and descriptions presented in this article bring together learning from social norms theory and religions and development research to meld a framework that can be used in context analysis for interventions with faith actors on child marriage.

We recommend this diagram be used by practitioners in their design and implementation of interventions around child marriage. It has been created particularly for design and planning stages of interventions when processes including formative research and context analysis may be ongoing to understand the landscape and identify which entry points and which types of intervention will be most impactful. For example, the framework could be used to help structure a dialogue between program staff, researchers, and faith actors to identify which of the behavioral drivers represent the most pressing issues in the intervention area and pinpoint the role of religion in the behaviors. If it is identified that religious leaders and faith communities in an area believe child marriage to be supported by their interpretation of religious texts, then an appropriate intervention would focus on theological interpretation with religious scholars and trainings with religious leaders. The framework can equally help identify the times in which religion is not an influential aspect of child marriage. When the predominant influence is the drive for economic security potentially provided by marriage, religious influence may not play as strong a role. With more analysis, intervention designers can further use the framework to understand how these behavioral drivers interact with each other. For example, information from formative research could demonstrate that religious leaders are performing child marriage ceremonies both because of their role as the leader of the religious community with the expectation that they perform all marriage ceremonies and because they receive a financial benefit for performing ceremonies. Therefore, the intervention design needs to address both aspects in its engagement with religious communities. The framework seeks both to demonstrate the complexity of religious engagement (i.e., interventions with aspects of religious engagement need to be tailored to the context, and there is not a 1-stop-shop model of religious engagement) while also providing guidance to practitioners as they navigate this complexity by laying out the common issues they might encounter and how to analyze and understand these issues within the structure of social norms. While we have presented this framework on child marriage, a similar review of literature and adaptation of the framework could make it relevant to any topic in which social and behavioral changes are the goal.

Intervention designers can further use the framework to understand how these behavioral drivers interact with each other.

In the context of the larger FPCC project, this analysis of drivers is operationalized in a process called Mind-Heart Dialogue^30^^,^^31^ that uses participatory methods to help faith and development actors understand the root causes of religiously influenced harmful social norms in their communities. The framework outlined in this article can be used in conjunction with the FPCC Program Guidance,^30^ which outlines a program cycle with a series of key intervention points for improved religious engagement in social and behavior change work. An early part of the program cycle prioritizes “learning and listening to children and their communities” and emphasizes the need to conduct formative research to thoroughly understand the religious drivers present within a community. The application of the diagram in this article is particularly important at this stage of the process when it can be used to help identify which religious drivers are prominent in the community in question. This then leads to proper identification of the best entry points and intervention sites (such as parent and marriage counseling, peer-to-peer support within faith communities, faith meetings/celebrations/retreats, rituals and rites of passage, faith-connected government services, and faith-based media).

Working with faith actors on social and behavior change work across child well-being areas means building long-term and non-instrumentalizing relationships. With reference to deeply rooted and complex social norms, such as child marriage, any strategy to engage faith actors requires long-term commitment and a deep understanding of the religious dynamics in that context. Research and analysis are key to understanding the complexity of religious dynamics in a community. This framework can aid practical analysis of the religious drivers of child marriage to further and deepen practitioners’ understanding.

## Notes

**Peer Reviewed**

**First Published Online:** April 15, 2024.
